# Morphological and Structural Transformations of Fe-Pd Powder Alloys Formed by Galvanic Replacement, Annealing and Acid Treatment

**DOI:** 10.3390/ma15103571

**Published:** 2022-05-17

**Authors:** Sergey A. Petrov, Dina V. Dudina, Arina V. Ukhina, Boris B. Bokhonov

**Affiliations:** Institute of Solid State Chemistry and Mechanochemistry SB RAS, Kutateladze Str. 18, 630128 Novosibirsk, Russia; petrov@solid.nsc.ru (S.A.P.); dina1807@gmail.com (D.V.D.); auhina181@gmail.com (A.V.U.)

**Keywords:** core–shell particles, Fe-Pd alloys, galvanic replacement, selective dissolution, hollow particles, Mössbauer spectroscopy

## Abstract

In this article, we report the preparation and structural features of Fe-Pd powder alloys formed by galvanic replacement, annealing and selective dissolution of iron via acid treatment. The alloys were studied by the X-ray diffraction phase analysis, Mössbauer spectroscopy, scanning electron microscopy, and energy-dispersive spectroscopy. The Fe@Pd core–shell particles were obtained by a galvanic replacement reaction occurring upon treatment of a body-centered cubic (bcc) iron powder by a solution containing PdCl_4_^2−^ ions. It was found that the shells are a face-centered cubic (fcc) Pd(Fe) solid solution. HCl acid treatment of the Fe@Pd core–shell particles resulted in the formation of hollow Pd-based particles, as the bcc phase was selectively dissolved from the cores. Annealing of the Fe@Pd core–shell particles at 800 °C led to the formation of fcc Fe-Pd solid solution. Acid treatment of the Fe-Pd alloys formed by annealing of the core–shell particles allowed selectively dissolving iron from the bcc Fe-based phase (Fe(Pd) solid solution), while the fcc Fe-rich Fe-Pd solid solution remained stable (resistant to acid corrosion). It was demonstrated that the phase composition and the Fe/Pd ratio in the alloys (phases) can be tailored by applying annealing and/or acid treatment to the as-synthesized Fe@Pd core–shell particles.

## 1. Introduction

Particulate materials of different morphologies based on noble metals exhibit properties promising for practical applications. Pd-, Au- and Pt-based materials attract a lot of attention, as indicated by a large number of publications on their preparation and properties [1,2]. Noble metals are promising for electrocatalysis [3], electrochemical biosensors [4], energy conversion/storage devices, and fuel cells [5]. Separate particles of noble metals and porous materials are obtained [6]. The cost of the noble metals is a factor significantly limiting their large-scale application [7,8]. Alloying with base metals allows reducing the consumption of the noble metals. Studies show that the catalytic activity of alloys containing inexpensive (base) metals can be higher than that of the pure (unalloyed) noble metals [6,9].

Among a variety of noble metal-based particles and porous alloys, Fe-Pd alloys are of particular interest. The Fe-Pd alloys show the following functional properties: catalytic activity [10,11], magnetic shape memory (for the Fe_70_Pd_30_ composition) [12], surface-enhanced Raman scattering [13], and hydrogen absorption (Pd-rich alloy) [14,15]. Fe/Pd particles showed an electrocatalytic effect when compared with Pd [16]. The properties of Fe-Pd alloys depend on their composition and structural characteristics [17,18,19,20].

The Fe-Pd alloys can be prepared by different methods. A porous PdFe alloy was fabricated through one-step mild dealloying of a PdFeAl ternary source alloy in NaOH solution [21]. Thin films of Pd were obtained by the selective dissolution of iron in HCl solution from Fe_75_Pd_25_ films deposited on a gold-coated silicon oxide substrate [13,22]. In ref. [20], a Fe-Pd alloy was formed by combining electrodeposition and selective dissolution. Pd/Fe wire electrodes were prepared via a galvanic replacement reaction between tetrachloropalladate (PdCl_4_^2−^) and a Fe wire [23]. Fe-Pd alloy particles were obtained by reducing a mixture of iron and palladium compounds by sodium borohydride [24,25]. In ref. [26], monodisperse FePd particles were prepared by polyol reduction of palladium acetylacetonate and thermal decomposition of iron pentacarbonyl. It was shown that particles of Fe_35_Pd_65_ can be obtained via the thermal decomposition of metal–organic compounds of iron and palladium [27]. The Fe-Pd alloys can also be produced by physical methods. For example, Fe@Pd, Fe@Pt, and Fe@Au core–shell particles supported by silicon carbide were prepared by plasma sputtering deposition [28].

Galvanic replacement is a promising method of the formation of metallic coatings and preparation of core–shell structures [29,30,31,32,33]. This method is quite simple from the technological perspective. In this process, particles/objects of less noble metals of different sizes and morphologies are used as templates to tailor the structural characteristics of the resultant materials enriched with a more noble metal. The selective dissolution of less noble metals from the binary alloys obtained by galvanic replacement allows forming hollow particles and porous materials. In this manner, porous Au, Pt, Pd, Ru, Cu and Ni can be produced by dissolving a less noble metal from the corresponding alloys.

Fe-Pd powders have been obtained by galvanic replacement reaction starting from iron nanocubes as templates [16]. To the best of our knowledge, no study has been conducted on the formation of Fe-Pd alloys by galvanic replacement reaction using micrometer-sized iron templates. These templates are widely available as commercial powders. In the present work, we investigated the morphological and phase transformations occurring upon galvanic replacement of iron by palladium, annealing of the products and selective dissolution of iron from the synthesized powder alloys. The goal of the study was to trace the structural evolution in the synthesized alloys and determine factors responsible for the structural changes.

## 2. Materials and Methods

Fe@Pd core–shell microparticles were synthesized by galvanic replacement reaction. Carbonyl iron (99.9%, “SyntezPKZh”, Dzerzhinsk, Russia) was used as a reactant and a template.

The iron powder was placed in a solution of PdCl_2_ in HCl acid for 3 min. Then, the solution was filtrated, and the residue was washed with deionized water several times and dried at room temperature. The concentration of PdCl_2_ in solution was selected such that ensured a Fe/Pd atomic ratio in the product of galvanic replacement of 4:1 upon full reduction of palladium ions.

The product of galvanic replacement was annealed in vacuum at 800 °C for 30 min.

Acid treatment of the as-synthesized core–shell particles and annealed powders was conducted in 35% HCl solution.

X-ray diffraction (XRD) patterns of the samples were recorded using a D8 ADVANCE diffractometer (Bruker AXS, Karlsruhe, Germany) with Cu Kα radiation. The crystallite sizes and the lattice parameters of the phases were calculated in TOPAS 4.2 software (Bruker AXS) by conducting Rietveld refinement of the experimentally recorded profiles.

Mössbauer spectroscopy (MS) was carried out on a NP 255/610 spectrometer (Budapest, Hungary) with a ^57^Co(Rh) source.

The morphology of the particles was studied by scanning electron microscopy using a S-3400 N microscope working at 30 kV (Hitachi, Tokyo, Japan). The elemental analysis of the alloys was carried out using an energy-dispersive spectroscopy (EDS) unit (NORAN Spectral System 7, Thermo Fisher Scientific Inc., Waltham, MA, USA) attached to the microscope. The initial iron particles and the synthesized core–shell particles were mounted into resin and polished to observe their cross-sectional structure.

## 3. Results and Discussion

### 3.1. Structural Characterization of the Iron Powder

**Figure 1:**
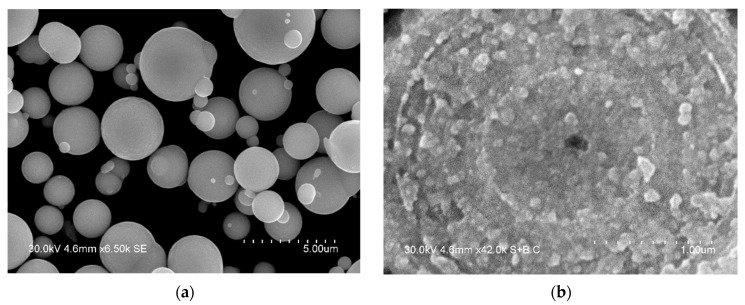
Morphology (**a**) and internal structure (**b**) of the carbonyl iron particles.

**Figure 2:**
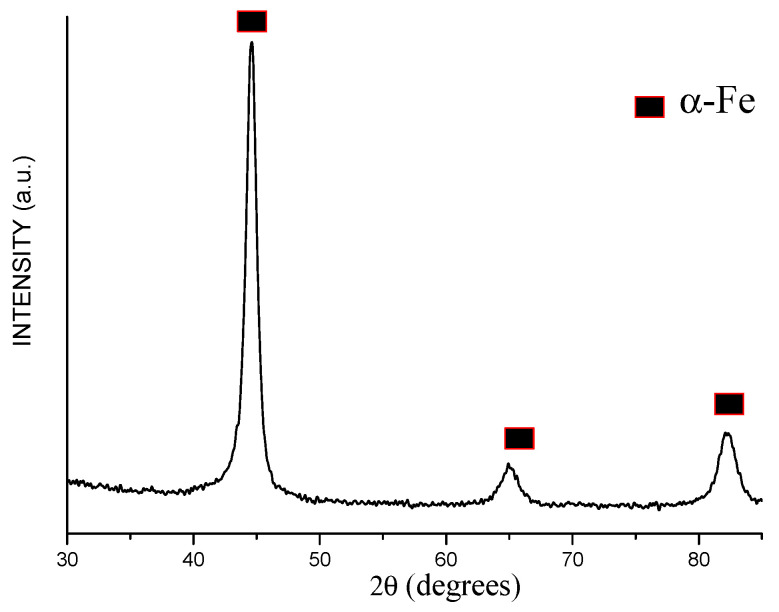
X-ray diffraction (XRD) pattern of the carbonyl iron powder.

The particles of the iron powder used in the present work had a spherical shape (Figure 1a). The structure of the particles was revealed by etching their polished cross-sections by a HNO_3_-C_2_H_5_OH mixture. The multiple layers form an onion structure, each shell consisting of nanosized particles (Figure 1b). The XRD peaks of the powder (Figure 2) are those of body-centered cubic (bcc) iron (α-Fe). The peaks are significantly broadened. The crystallite size of iron was calculated to be 10 ± 1 nm. The lattice parameter of iron was determined to be 2.868 ± 0.001 Å, which is close to the lattice parameter of pure iron (PDF card 6-696).

The phase transformations in the Fe-Pd materials obtained in the present work (based on results of the XRD analysis) and the lattice parameters of the phases are presented in Table 1.

**Figure 3:**
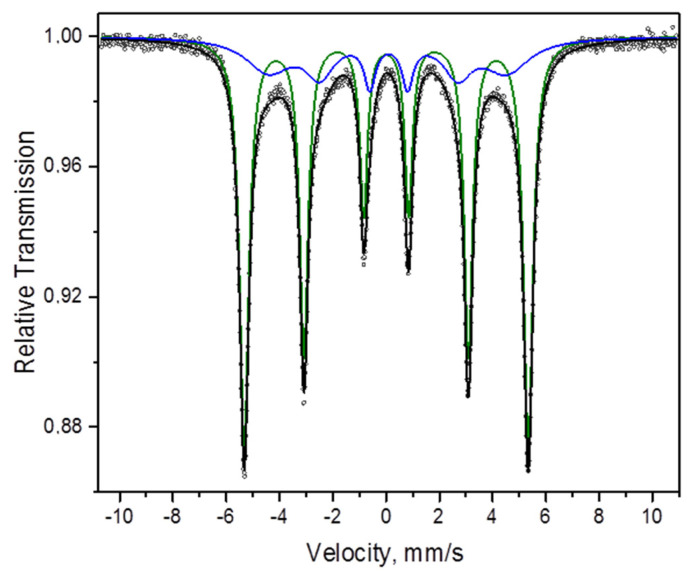
Mössbauer spectrum of the carbonyl iron powder: dots—experimental data, black line—spectrum represented as a superposition of two sextets (green and blue lines).

The Mössbauer spectrum of the carbonyl iron powder can be described as a superposition of two sextets (Table 2, Figure 3). The first sextet is due to the presence of α-Fe (Figure 3, green line). The parameters of the second sextet (Figure 3, blue line) suggest that it can originate from “disordered” α-Fe (the presence of multiple defects in the crystalline lattice of α-Fe), which agrees well with the results of XRD and microscopy investigations of the structure of this powder.

### 3.2. Formation of Fe@Pd Core–Shell Particles by Galvanic Replacement

In order to deposit palladium on the surface of iron particles, a galvanic replacement reaction was carried out. This reaction occurred upon treatment of the iron particles by PdCl_2_ solution in HCl acid. The following scheme describes the chemical processes upon galvanic replacement:

PdCl_4_^2−^ + 2e → Pd^0^ + 4Cl^−^

Fe^0^ → Fe^2+^ + 2e

Fe + PdCl_4_^2−^ → Pd^0^ + Fe^2+^ + 4Cl^−^

**Figure 4:**
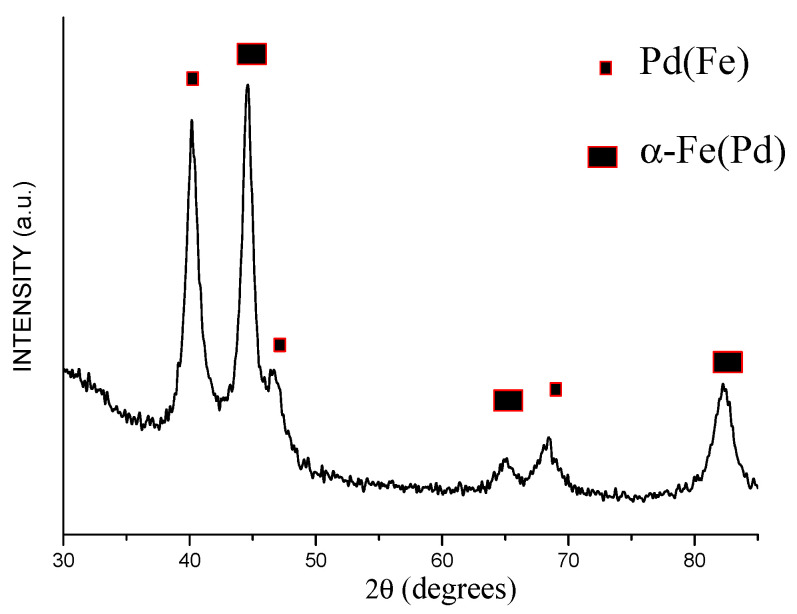
XRD pattern of the Fe@Pd core–shell particles obtained by galvanic replacement reaction.

On the XRD pattern of the product of galvanic replacement, broadened reflections with positions close to those of metallic palladium (face-centered cubic (fcc) phase) are detected, along with reflections of α-Fe (Figure 4). The lattice parameter of the fcc phase was calculated to be 3.879 ± 0.002 Å (Table 1), which is lower than the lattice parameter of pure palladium (3.890 Å, PDF card 46-1043). This can be explained by the dissolution of iron in the palladium lattice (formation of Pd(Fe) solid solution) during the deposition of palladium on the iron surface (the atomic radius of iron is smaller than that of palladium). The crystallite size of the fcc was determined to be 7 ± 1 nm. The crystallite size of the bcc phase of the product of galvanic replacement was 11 ± 1 nm (did not change relative to the untreated iron powder). The lattice parameter of the bcc phase (2.873 ± 0.002 Å) was increased relative to pure iron (Table 1).

**Figure 5:**
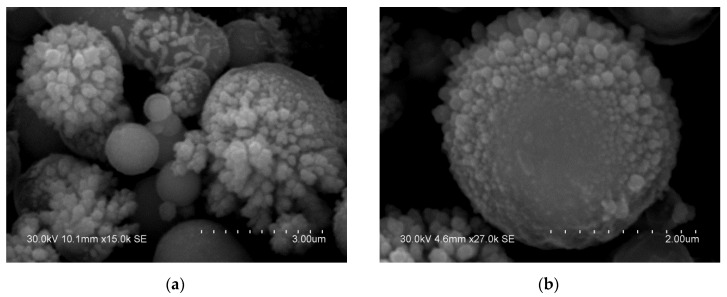

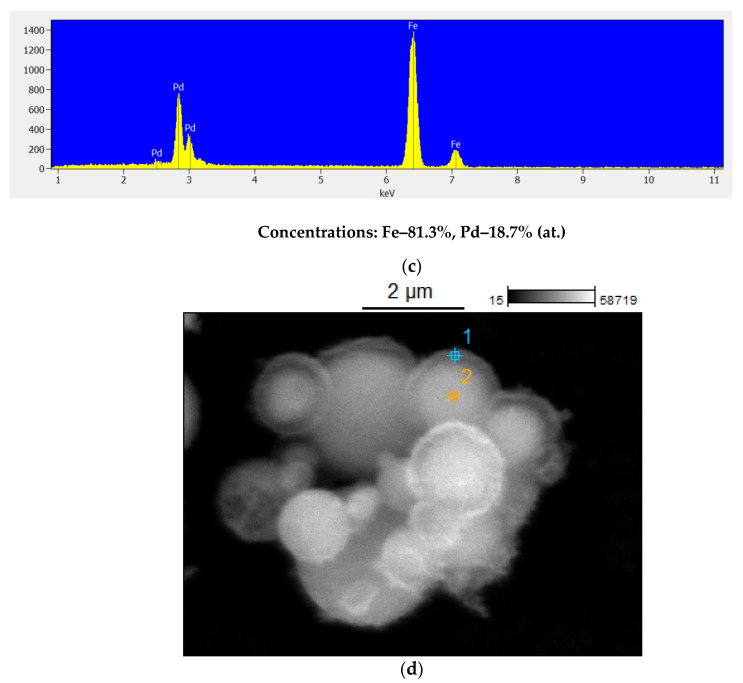
(**a**,**b**) Morphology of the Fe@Pd core–shell particles obtained by galvanic replacement reaction, different magnifications, (**c**) energy-dispersive spectroscopy (EDS) of the Fe@Pd core–shell particles obtained by galvanic replacement reaction, (**d**) cross-section of the core–shell particles (concentration of Pd in point 1 is higher than in point 2, as determined by EDS).

The electron microscopy images of the product of galvanic replacement show that a layer is formed on the surface of the iron particles (Figure 5a,b). This layer consists of separate submicron particles. The product of galvanic replacement is Fe@Pd core–shell particles, in which the core is Fe-based and the shell is Pd-based. It should be noted that some shells are not continuous and have orifices in them, which is typical for particles obtained by this method. The EDS analysis (Figure 5c) shows that the Fe/Pd atomic ratio in the product of galvanic replacement is close to 4:1, which indicates a nearly full reduction of palladium ions present in the solution by metallic iron. In Figure 5d, cross-sections of the core–shell particles are shown. The presence of shell is clearly visible in the image.

**Figure 6:**
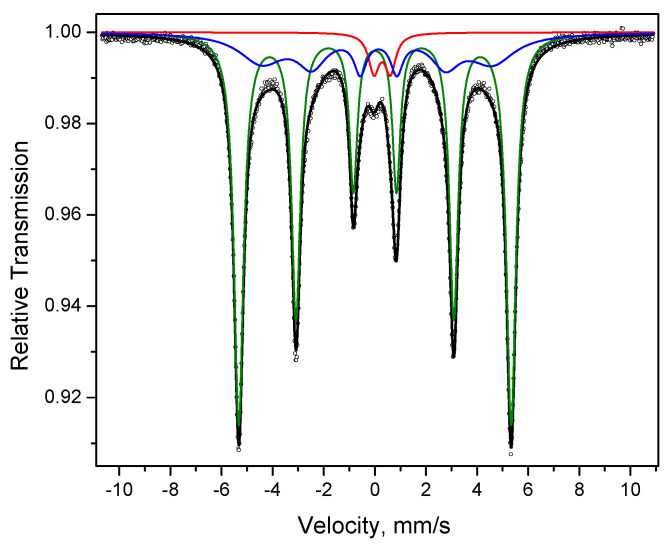
Mössbauer spectrum of the Fe@Pd core–shell particles obtained by galvanic replacement reaction: dots—experimental data, black line—spectrum represented as a superposition of two sextets (green and blue lines) and a doublet (red line).

In the Mössbauer spectrum of the core–shell particles, two sextets are present corresponding to α-Fe (green line) and α-Fe with a “disordered” structure (blue line), as discussed in Section 3.1 (Figure 6). In addition, a doublet is present (red line), which can be related to the presence of Fe^3+^. The presence of this doublet can be due to the formation of a small amount of the oxide phase during the preparation of the sample. A similar effect was earlier observed in ref. [34].

### 3.3. Selective Dissolution of Iron from the Fe@Pd Core–Shell Particles: Formation of Hollow Palladium Particles

**Figure 7:**
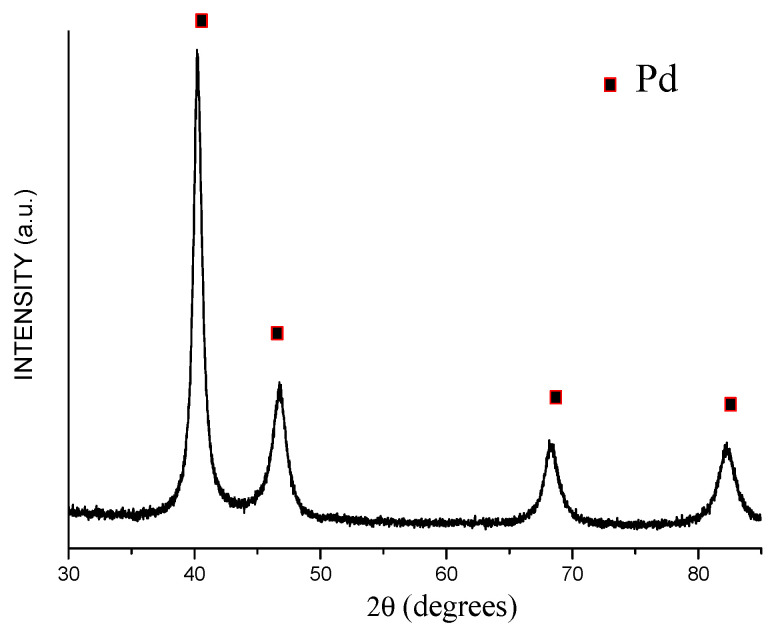
XRD pattern of the product of acid treatment of Fe@Pd core–shell particles (hollow particles of a Pd-based alloy).

Upon treatment of the Fe@Pd core–shell particles by HCl solution, iron was selectively dissolved. On the XRD patterns of the product of acid treatment, only reflections of palladium are seen (Figure 7). The crystallite size of the palladium is 11 ± 1 nm. The lattice parameter of palladium is 3.889 ± 0.001 Å, which is close to the lattice parameter of pure palladium.

**Figure 8:**
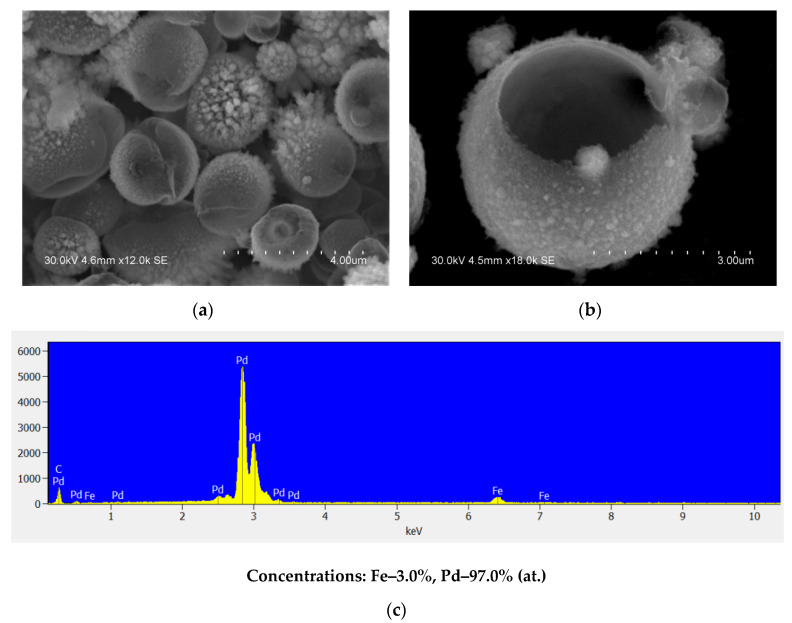
(**a**,**b**) Morphology of the product of acid treatment of Fe@Pd core–shell particles (hollow particles), different magnifications, (**c**) EDS of the product of acid treatment of Fe@Pd core–shell particles (hollow particles of the Pd-based alloy).

The electron microscopy images show that the acid treatment of the core–shell particles leads to the formation of hollow particles of palladium (Figure 8a,b). While the lines of the iron phase are absent from the XRD profile of the hollow particles, the analysis conducted by EDS shows the presence of iron (Figure 8c). The Fe/Pd atomic ratio in the shells was found to be equal to 1:32.

**Figure 9:**
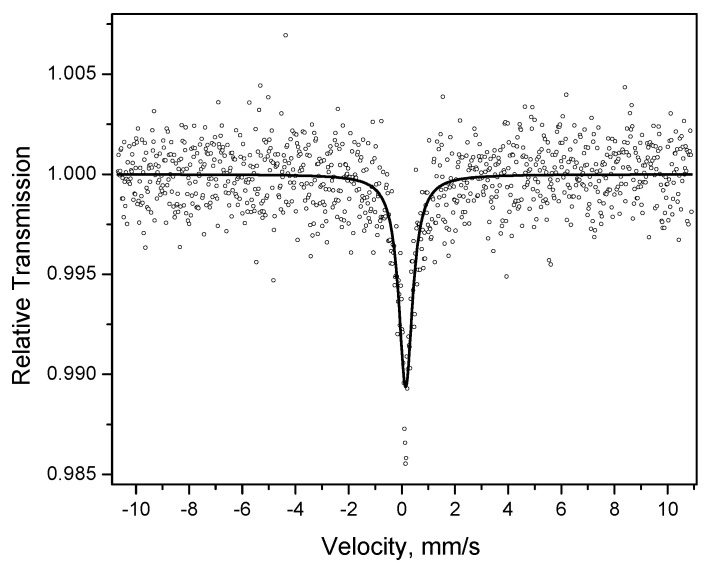
Mössbauer spectrum of the product of acid treatment of Fe@Pd core–shell particles (hollow particles): dots—experimental data, black line—spectrum (singlet).

The Mössbauer spectrum of the hollow particles is a broad singlet with an isomer shift of 0.15 mm s^−1^ (Figure 9), which is close to the isomer shift of iron dissolved in palladium (0.17 mm s^−1^) [35]. This indicates the presence of iron dissolved in palladium (the formation of Pd(Fe) alloy hollow particles).

### 3.4. Phase and Structural Evolution of the Fe@Pd Particles upon Annealing

The Fe-Pd phase diagram is rather complex [36,37]. There exist two intermetallic compounds (FePd and FePd_3_) in the system. Above 800 °C, the metals are fully mutually soluble. Below 200 °C, Fe_x_Pd_100−x_ solid solutions of fcc structure form at 0 < x < 10. When x > 70, solid solutions of bcc structure form.

**Figure 10:**
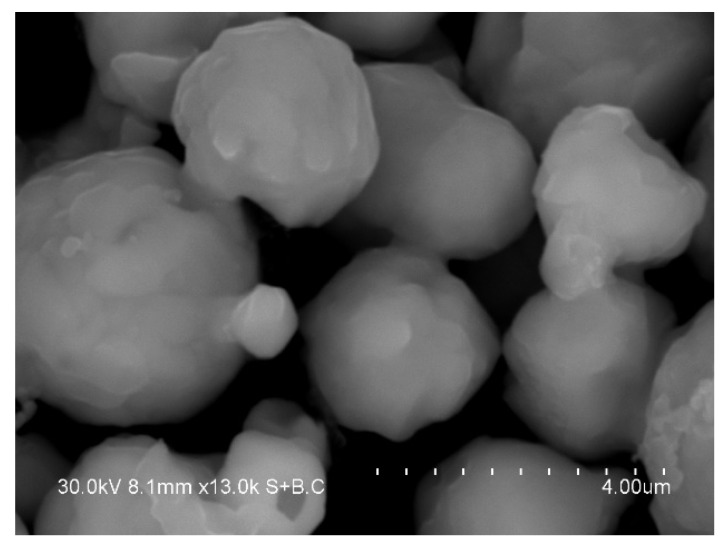
Morphology of the powder alloy obtained by annealing of the Fe@Pd core–shell particles.

**Figure 11:**
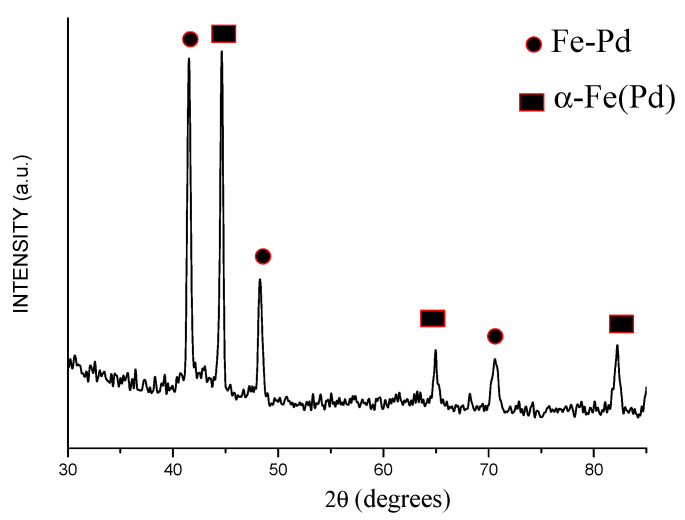
XRD pattern of the powder alloy obtained by annealing of the Fe@Pd core–shell particles (Fe-Pd is a Fe-rich solid solution).

The electron microcopy investigations showed that the product of annealing of the core–shell Fe@Pd particles at 800 °C consists of dense particles 1–4 μm in size (Figure 10). The XRD pattern of the annealed powder showed the peaks of a bcc phase and a fcc phase (Figure 11). The latter shifted to higher angles relative to their positions on the pattern of the core–shell particles (Figure 4). The crystallite size of the metals increased after annealing: the crystallite size of the bcc phase was 68 ± 18 nm, while that of the fcc phase was 64 ± 19 nm. The lattice parameter of the bcc phase was 2.875 ± 0.001 Å in the annealed powder. The calculated lattice parameter of the fcc phase was 3.773 ± 0.001 Å, which is smaller than the lattice parameter of metallic palladium (Table 1). A significant reduction in the lattice parameter of the fcc phase can be explained by the formation of a Fe-Pd solid solution during annealing of the Fe@Pd core–shell particles. The concentration of palladium in this solid solution is ≈49 at %, as calculated using Vegard’s rule. The formation of a solid solution with a close lattice parameter was described in [38] for a fcc structure of Fe_50_Pd_50_ composition. Thus, it can be concluded that annealing of the Fe@Pd core–shell particles activates diffusion and induces the formation of a Fe-rich fcc solid solution with a composition close to Fe_50_Pd_50_.

**Figure 12:**
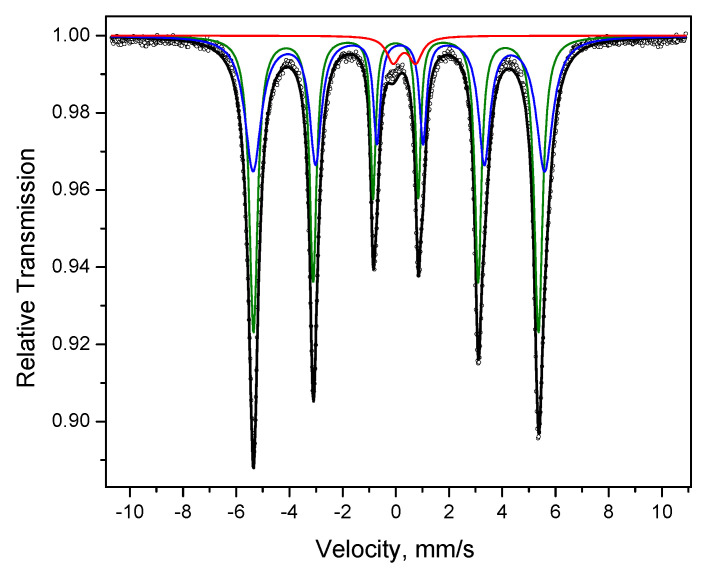
Mössbauer spectrum of the powder alloy obtained by annealing of the Fe@Pd core–shell particles: dots—experimental data, black line—spectrum represented as a superposition of two sextets (green and blue lines) and a doublet (red line).

The Mössbauer spectrum of the alloy obtained by annealing of the Fe@Pd core–shell particles shows two sextets (Figure 12). The parameters of one of them are close to those of α-Fe (green line), but the magnetic splitting is higher. The second sextet (blue line) has broadened lines, a greater isomer shift and even higher magnetic splitting. These characteristics suggest that the sextet is due to the presence of a Fe-rich Fe-Pd solid solution. In the spectrum, a doublet is present (red line), which can be related to the presence of Fe^3+^ in a low concentration, as noted above.

### 3.5. Selective Dissolution of Iron from the Annealed Alloy

**Figure 13:**
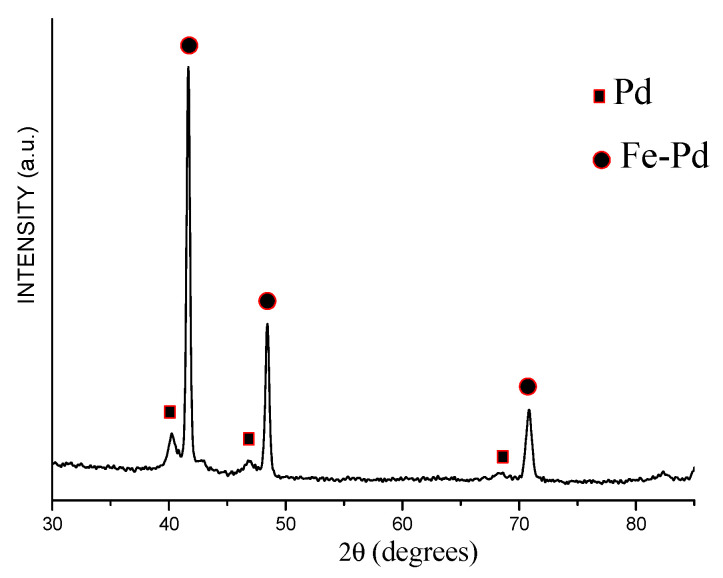
XRD pattern of the annealed powder treated in HCl solution (Fe-Pd is a Fe-rich solid solution).

**Figure 14:**
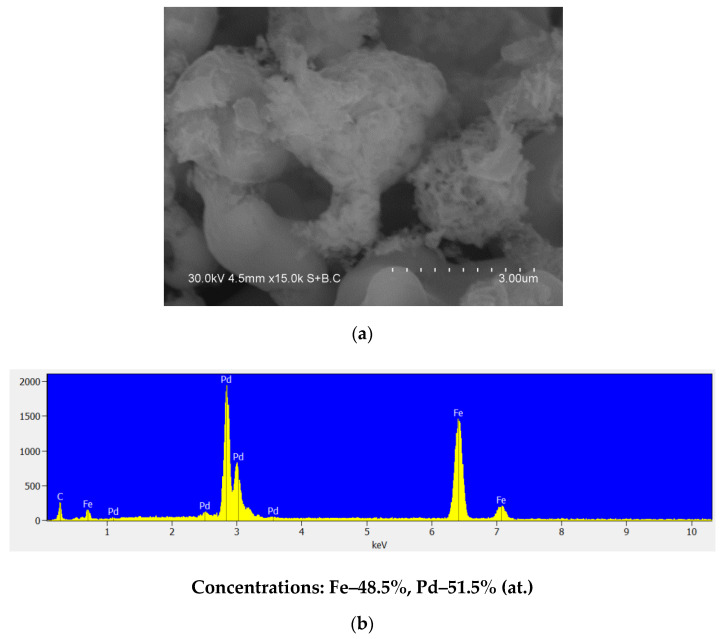
Morphology of the annealed powder treated in HCl solution (**a**), EDS of the annealed powder treated in HCl solution (**b**).

The treatment of the annealed powder in HCl solution led to the dissolution of iron from the bcc phase, as confirmed by the XRD analysis. No reflections of iron were detected (Figure 13). After HCl treatment, the alloy was composed of a Fe-Pd solid solution with a lattice parameter of 3.773 ± 0.001 Å (major phase) and metallic palladium with crystallites of 10 ± 2 nm (minor phase). The fcc Fe-Pd solid solution formed during annealing remained stable during the acid treatment (Table 1). The appearance of reflections of palladium is most likely due to the release of palladium upon the dissolution of iron from the bcc Fe(Pd) solid solution. The morphology of the particles can be seen in Figure 14a. The overall composition of the system changes from the Fe/Pd atomic ratio of 4:1 before the acid treatment to 1:1 after the acid treatment (Figure 14b). So, the acid treatment leads to the Pd enrichment of the system as a whole.

**Figure 15:**
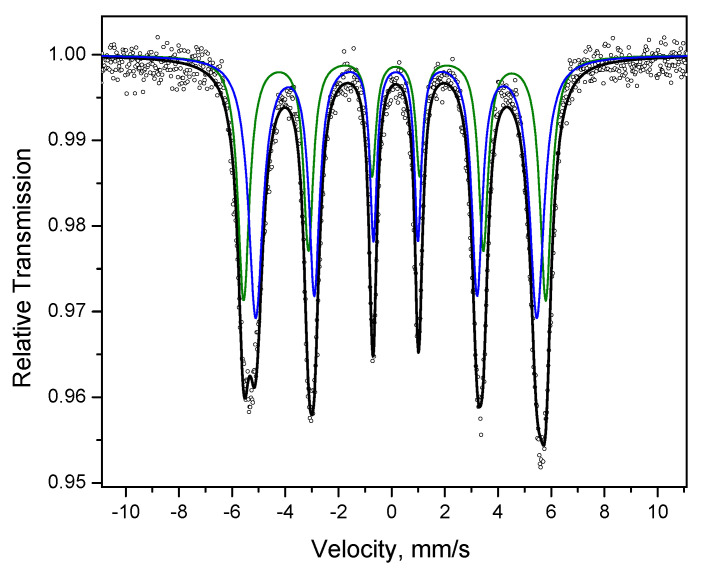
Mössbauer spectrum of the annealed powder treated in HCl solution: dots—experimental data, black line—spectrum represented as a superposition of two sextets (green and blue lines).

The Mössbauer spectrum of the powder after annealing and treatment in HCl solution (Figure 15) shows two sextets with parameters characteristic of Fe-rich Fe-Pd solid solutions marked in Table 2 as (I) and (II). This confirms that the solid solution was resistant to acid treatment and was preserved in the system. It should be noted that the acid treatment of the as-synthesized Fe@Pd core–shell particles and annealed powder produces different results. While it is possible to dissolve iron from the as-synthesized core-shell particles almost fully, the Fe-rich Fe-Pd solid solution formed upon annealing remains stable to the acid attack.

## 4. Summary

The morphological and structural features of Fe-Pd alloys formed by galvanic replacement, annealing and acid treatment have been studied. As galvanic replacement occurred, iron was replaced by palladium, which resulted in the formation of Fe@Pd core–shell microparticles. Based on results of XRD and Mössbauer spectroscopy, it was concluded that the Pd-based shells were a fcc Pd(Fe) solid solution. HCl acid treatment of the Fe@Pd core–shell particles allowed selectively dissolving iron from the cores and forming hollow Pd(Fe) particles. Upon annealing of the Fe@Pd core–shell particles, a Fe-rich Fe-Pd solid solution formed, which was confirmed by both XRD and Mössbauer spectroscopy. HCl treatment of the Fe-Pd alloy formed by annealing of the core–shell particles led to the selective dissolution of iron from the Fe-based phase (bcc Fe(Pd) solid solution), while the Fe-rich Fe-Pd fcc solid solution remained stable during the acid treatment. This work has shown than galvanic replacement and selective dissolution can be used for obtaining Fe-Pd alloys in the form of core–shell and hollow particles. Annealing and acid treatment can be applied to modify the phase composition and the ratio of metals in these alloys.

## Figures and Tables

**Table 1:** Phases (as determined by X-ray diffraction) and their lattice parameters (Å) of the Fe-Pd alloys described in the present work. The lattice parameter of pure metallic palladium is 3.890 Å, pure metallic bcc iron—2.867 Å (bcc—body-centered cubic, fcc—face-centered cubic). Atomic radius of iron is 126 pm. Atomic radius of palladium is 137 pm.

Powder	Bcc Fe	Bcc Fe(Pd) Solid Solution	Fcc Pd	Fcc Pd(Fe)/ Fcc Fe-Rich Fe-Pd Solid Solution *
Fe powder	+ 2.868 ± 0.001	-	-	-
Fe@Pd core–shell particles	-	+ 2.873 ± 0.002	-	+ 3.879 ± 0.002
Hollow Pd-based particles	-	-	+ 3.889 ± 0.001	-
Product of annealing of Fe@Pd at 800 °C	-	2.875 ± 0.001	-	(#) 3.773 ± 0.001
Product of annealing of Fe@Pd at 800 °C after acid treatment	-	-	+ 3.889 ± 0.001	+ (#) 3.773 ± 0.001

* Data related to the Fe-rich Fe-Pd solid solutions are marked (#).

**Table 2:** Parameters of the Mössbauer spectra of the Fe-Pd powder alloys *.

Powder	State of Iron	Area Fraction, %	δ, mm s^−1^	B_hf_, T	Δ, mm s^−1^
Fe powder	α-Fe	71	0.005	33.22	0.001
	disordered α-Fe	29	0.086	27.50	−0.02
Fe@Pd core–shell particles	α-Fe	71	0.005	33.23	0.002
	Fe^3+^	4	0.287	-	0.64
	disordered α-Fe	25	0.116	28.14	−0.03
Hollow Pd-based particles	Pd(Fe)	100	0.154	-	-
Product of annealing of Fe@Pd at 800 °C	α-Fe	51	0.001	33.38	0.007
	Fe-Pd solid solution	46	0.135	34.15	−0.02
	Fe^3+^	3	0.323	-	0.84
Product of annealing of Fe@Pd at 800 °C after acid treatment	Fe-Pd solid solution (I)	43	0.138	35.40	−0.02
	Fe-Pd solid solution (II)	57	0.164	32.94	0.01

* δ is the isomer shift reflecting the chemical neighborhood of the resonant nucleus, B_hf_ is magnetic hyperfine field, Δ is a measure of the distortion of the nearest neighborhood with respect to a cubic one.
